# The Mouse Cytomegalovirus Gene m42 Targets Surface Expression of the Protein Tyrosine Phosphatase CD45 in Infected Macrophages

**DOI:** 10.1371/journal.ppat.1006057

**Published:** 2016-12-07

**Authors:** Nadine Thiel, Kirsten A. Keyser, Niels A. W. Lemmermann, Jennifer D. Oduro, Karen Wagner, Carina Elsner, Anne Halenius, Tihana Lenac Roviš, Melanie M. Brinkmann, Stipan Jonjić, Luka Cicin-Sain, Martin Messerle

**Affiliations:** 1 Institute of Virology, Hannover Medical School, Hannover, Germany; 2 Institute of Virology, University Medical Center of the Johannes Gutenberg University, Mainz, Germany; 3 Helmholtz Centre for Infection Research, Braunschweig, Germany; 4 Institute of Virology, Medical Center, University of Freiburg, Freiburg, Germany; 5 Department of Histology and Embryology, Faculty of Medicine, University of Rijeka, Rijeka, Croatia; University of Wisconsin-Madison, UNITED STATES

## Abstract

The receptor-like protein tyrosine phosphatase CD45 is expressed on the surface of cells of hematopoietic origin and has a pivotal role for the function of these cells in the immune response. Here we report that following infection of macrophages with mouse cytomegalovirus (MCMV) the cell surface expression of CD45 is drastically diminished. Screening of a set of MCMV deletion mutants allowed us to identify the viral gene m42 of being responsible for CD45 down-modulation. Moreover, expression of m42 independent of viral infection upon retroviral transduction of the RAW264.7 macrophage cell line led to comparable regulation of CD45 expression. In immunocompetent mice infected with an m42 deletion mutant lower viral titers were observed in all tissues examined when compared to wildtype MCMV, indicating an important role of m42 for viral replication *in vivo*. The m42 gene product was identified as an 18 kDa protein expressed with early kinetics and is predicted to be a tail-anchored membrane protein. Tracking of surface-resident CD45 molecules revealed that m42 induces internalization and degradation of CD45. The observation that the amounts of the E3 ubiquitin ligases Itch and Nedd4 were diminished in cells expressing m42 and that disruption of a PY motif in the N-terminal part of m42 resulted in loss of function, suggest that m42 acts as an activator or adaptor for these Nedd4-like ubiquitin ligases, which mark CD45 for lysosomal degradation. In conclusion, the down-modulation of CD45 expression in MCMV-infected myeloid cells represents a novel pathway of virus-host interaction.

## Author Summary

Human cytomegalovirus (HCMV) is a tenacious pathogen, which can be life-threatening for immunocompromised patients and immunologically immature newborns. The pathogenicity of HCMV is owed to a plethora of immunomodulatory functions that interfere with host defense mechanisms. Such viral functions can teach us about viral pathogenesis mechanisms, and also about the functioning of immune cells. In this study we report that the mouse cytomegalovirus (MCMV)–a close relative of HCMV–influences surface expression of the cellular protein CD45 on macrophages and we identified the viral gene m42 mediating this effect. CD45 has long been known to be essential for the functioning of lymphocytes, however, its role in macrophages is less well understood. Growth analysis of a viral mutant indicated that the m42 gene confers a replication advantage to MCMV *in vivo*. We found that the m42 protein induces internalization of CD45 from the plasma membrane and degradation in lysosomes—most likely triggered by interaction of m42 with a ubiquitin ligase. In our study we detected a new element in the complex interaction of cytomegaloviruses with host cells, and further investigation into this mechanism may provide us with new insights into the functions of CD45 in myeloid cells.

## Introduction

Cytomegaloviruses (CMVs) possess the largest genomes among the herpesviruses [1–3] and dedicate a substantial portion of their genomic coding capacity to accessory functions that are not directly needed for replication of the DNA genome or as structural components for the assembly of progeny virions [4,5]. Among the accessory genes in turn many encode products that interfere with various pathways of the host immune response (reviewed in [6–9]). The fact that CMVs encode so many immunomodulatory genes may be owed to the close relationship that these viruses established with myeloid cells such as macrophages and dendritic cells [10,11]. These cells are central for both initiating the innate immune response as well as for shaping adaptive immunity against infectious pathogens. Despite these adverse conditions CMVs managed to employ myeloid cells as targets for lytic replication [12–14], and moreover as safe harbor for latent infection and a site of reactivation [10,15–21]. Therefore, the development of numerous immunomodulatory functions was probably a necessity for CMV and may be the result of an extensive evolutionary virus-host arms race. In healthy individuals infection with human CMV (HCMV) is nevertheless usually well controlled by the immune system, particularly by NK cells and CD8 T cells, resulting in unapparent infection [22]. However, the balance between host defense and viral countermeasures is delicate, and consequently CMV infection can lead to severe and sometimes life-threatening manifestations in patients with a weakened immune system or in immunologically immature fetuses or newborns [22].

The competition with the host response forced the CMVs to dampen those host defense mechanisms that threaten to eliminate the viruses. Viral immunomodulatory proteins may therefore teach us about yet unknown aspects of immune defense pathways and can point to Achilles’ heels of the host defense, thereby defining targets for potential therapeutic intervention. Although the best studied CMV members–mouse and human CMV (MCMV and HCMV)–underwent separate co-evolution with their respective hosts over almost 100 million years, they frequently developed strategies to interfere with similar processes of the immune system of mouse and man. Interestingly, gene products of MCMV and HCMV that target the same host pathway often display little sequence similarity and sometimes even their mode of action is different, indicating convergent evolution. Thus, by investigating immunomodulatory proteins of MCMV and HCMV and by comparing their function and working mechanism, we may learn about new host defense processes.

We have recently discovered that the HCMV UL11 protein can bind to the host protein CD45 [23], a protein tyrosine phosphatase expressed on the surface of most cells of hematopoietic origin, including T and B lymphocytes, NK cells, dendritic cells and macrophages [24]. CD45 is a positive regulator of antigen receptor signaling, which has been intensively studied and found to be central for the development and activation of T cells, B cells and NK cells (for review see refs. [25,26]). Consequently, disruption of the CD45 gene results in severe combined immunodeficiency in man and mouse [27–32]. Major substrates of the CD45 phosphatase are Src family kinases (SFKs), e.g. Lck in T cells; the removal of a phosphate group from an inhibitory tyrosine residue in the C-terminal domain leads to a primed state of SFKs, enabling them to transduce signals received by receptors to which they are associated. Dephosphorylation of a tyrosine in the autocatalytic domain abrogates the activity of SFKs and explains the negative regulatory role of CD45 observed under certain conditions. Studies with myeloid cells from CD45-deficient mice point to an involvement of CD45 with a number of different functions in these cells, ranging from proliferation upon stimulation with growth factors over adhesion and migration [33,34] to Toll-like receptor (TLR) signaling and production of type I interferons and other cytokines [35–39]. While some of these functions can be explained by the known axis between CD45 and SFKs, the mechanistic basis for other functions, e.g., the impact on TLR signaling, remains elusive [26,40].

Since the functional consequences of the interaction between the HCMV protein UL11 and CD45 are difficult to assess for HCMV infection [41], we wondered whether MCMV has evolved a similar mechanism aiming at CD45. Most viral immunomodulatory proteins exert their function directly within infected cells, and therefore we focused in this study on target cells of MCMV infection that express CD45, in particular on macrophages. We report that MCMV leads to diminished CD45 surface expression on these cells. Furthermore, we found that the viral gene m42 mediates this effect and analyzed the mechanisms of CD45 down-modulation. Infection experiments with an MCMV Δm42 mutant revealed that already an early step of the replication process *in vivo* is affected.

## Results

### MCMV infection leads to diminished CD45 cell surface expression in macrophages

**Fig 1:**
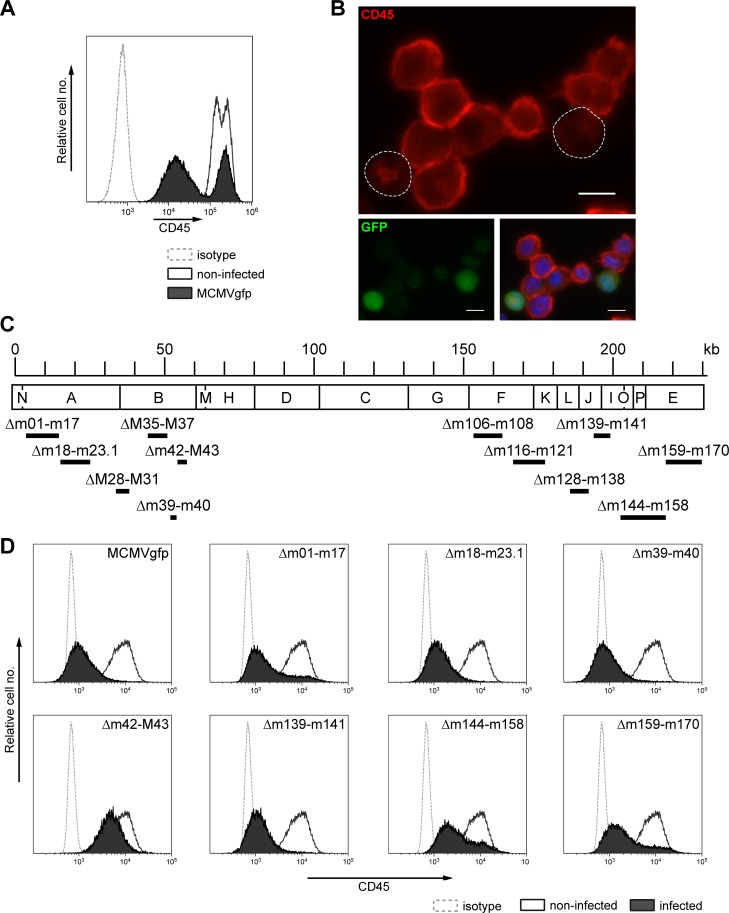
CD45 surface expression is reduced in MCMV-infected RAW264.7 macrophages. (A) RAW264.7 cells were either mock infected (open histogram) or infected with MCMVgfp (filled histograms) at an MOI of 3. 24 h p.i. CD45 surface expression was determined by flow cytometry for all cells of the cultures, except dead cells, which were excluded based on 7-AAD staining. Dotted line, isotype control. (B) Localization of CD45 was assessed 24 h p.i. by fluorescence microscopy in uninfected and infected (GFP+) RAW264.7 cells that were fixed, permeabilized and immunostained with a CD45-specific Ab. Cell nuclei were counterstained with Hoechst dye. Scale bars, 10 μm. (C) Schematic representation of the 230-kb MCMV genome (HindIII map), indicating the genes lacking in the respective deletion mutants. (D) RAW264.7 cells were mock-infected (open histograms) or infected (filled histograms) with the indicated deletion mutants, and 24 h p.i. immunostained to analyze CD45 surface levels. Dotted line, isotype control. For (D) gating was on living cells and for samples with infected cells additionally on GFP^+^ cells.

During our previous studies when we investigated the immune response against MCMV in lungs of neonatal mice [42,43], we noticed that infected macrophages displayed less staining with CD45 antibodies than non-infected macrophages. To investigate the putative interference of MCMV with CD45 expression in more detail, we infected RAW264.7 macrophages with a GFP-expressing MCMV strain (MCMVgfp) and examined the cells 24 h post infection (p.i.) by flow cytometry. In infected cells the amount of CD45 present at the cell surface was substantially reduced (Fig 1A and S1A Fig). Inspection of infected cells by fluorescence microscopy confirmed that only residual amounts of CD45 remained at the plasma membrane (Fig 1B). Comparable results were obtained upon infection of the dendritic cell line DC2.4 (S1D Fig) and bone-marrow-derived macrophages, and also when wildtype MCMV (MCMVwt; devoid of the GFP marker) was used for infection. Treatment of RAW264.7 cells with UV-inactivated virus did not affect CD45 expression (S1C Fig). We therefore supposed that an MCMV-encoded factor mediates down-regulation of CD45 in infected macrophages and other antigen-presenting cells.

### The MCMV m42 gene is involved in modulating CD45 expression

**Fig 2:**
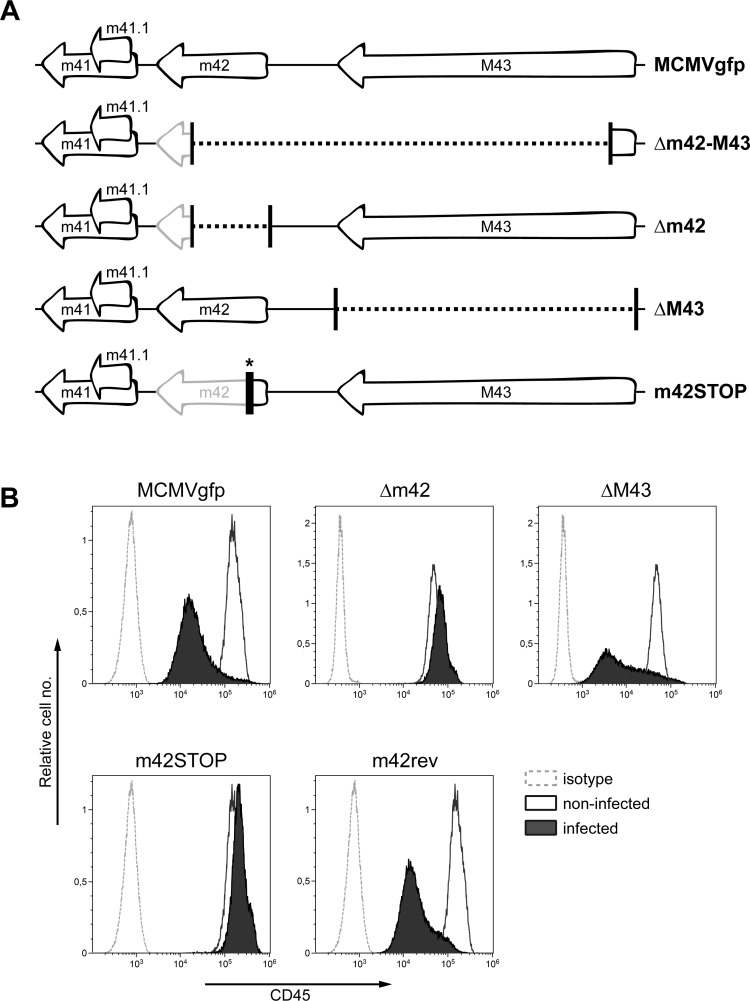
The MCMV ORF m42 is involved in CD45 down-regulation. (A) (top) Schematic representation of the genomic region harboring ORFs m41 to M43. (bottom) Sequences missing in the indicated deletion mutants are depicted by dotted lines. ORFs that were disrupted (and remaining parts of ORFs) are labelled in gray. The bar with the asterisk denotes a stop cassette disrupting the m42 ORF. (B) RAW264.7 cells were infected with the indicated viruses and GFP-positive cells were analyzed for CD45 surface expression as described in Fig 1.

In order to identify the viral gene responsible for the observed phenotype, we made use of a set of MCMV deletion mutants (Fig 1C) that lack various parts of the viral genome, covering most genes with accessory functions non-essential for viral replication in cell culture [44,45]. Following infection of RAW264.7 macrophages with the different mutants, CD45 levels were examined by flow cytometry one day later. The results obtained with selected mutants are depicted in Fig 1D. Except of the deletion mutant lacking ORFs m42 and M43, all other mutants led to strong down-modulation of CD45 expression. To assign the function to one of the two ORFs missing in the MCMVgfp-Δm42-M43 mutant, additional mutants were generated with a deletion in either ORF m42 or M43 only (Fig 2A). Infection experiments with these mutants revealed that only the MCMVgfp-Δm42 mutant displayed a loss-of-function phenotype (Fig 2B), strongly suggesting that a gene product encoded by the m42 ORF is involved in the regulation of CD45 surface expression. However, since several transcripts spanning this region have been reported [46,47], a contribution of neighboring ORFs could not be excluded. Therefore, the MCMVgfp-m42STOP mutant was generated that carries only a short DNA cassette containing stop codons within ORF m42, preventing synthesis of a functional protein. Moreover, a rescuant with a restored m42 ORF was constructed based on the genome of the MCMVgfp-m42STOP mutant, to exclude that an accidental mutation elsewhere in the viral genome led to the phenotype. In line with the hypothesis that m42 is the candidate gene, the MCMVgfp-m42STOP mutant did not diminish CD45 surface expression, whereas the rescuant MCMVgfp-m42rev regained this function (Fig 2B).

### The Δm42 mutant replicates to lower titers *in vivo*

**Fig 3:**
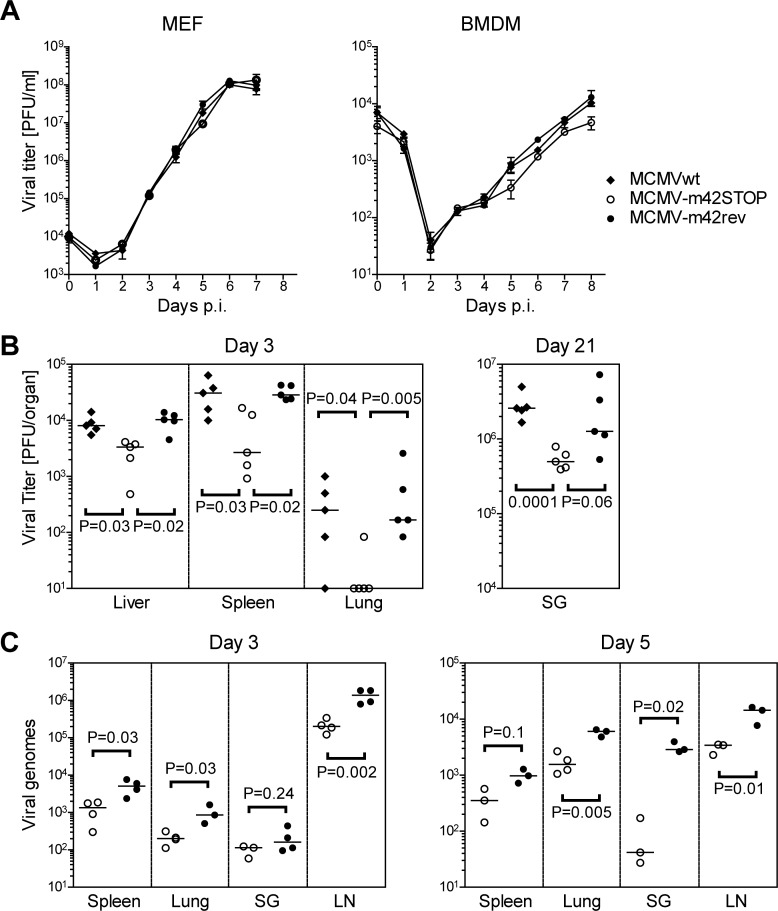
Growth of the m42 mutant *in vitro* and *in vivo*. (A) Murine embryonic fibroblasts (MEF) or bone marrow-derived macrophages (BMDM) were infected at an MOI of 0.1 with MCMVwt, MCMV-m42STOP or MCMV-m42rev. For the indicated time period cell culture supernatant was harvested daily and viral titers were determined by plaque assay on MEF. Each data point represents the average titer ± SD of triplicate cultures. (B) BALB/c mice were infected intraperitoneally with 2 × 10^5^ PFU of MCMVwt, MCMV-m42STOP or MCMV-m42rev. At the indicated time points viral titers of organ homogenates were determined by plaque assay on MEF. Data are representative of two independent experiments. (C) Viral DNA loads in organs of BALB/c mice infected with 2 × 10^5^ PFU via the footpad route with MCMV-m42STOP or MCMV-m42rev were measured on day 3 and 5 p.i. qPCR was performed for the viral gene M55 (encoding glycoprotein B) and data were normalized to 10^5^ cells by *pthrp*-specific qPCR. Each symbol corresponds to an individual mouse; horizontal bars indicate median values. *P* values < 0.05 were considered statistically significant.

Next, we examined the growth characteristics of the MCMV-m42STOP mutant. In murine embryonic fibroblasts the growth kinetics of the mutant, of MCMVwt and the rescuant were indistinguishable (Fig 3A), demonstrating that the m42 gene is not essential for replication and production of viral progeny in these cells. However, in bone marrow-derived macrophages–cells that express the CD45 protein targeted by m42 –growth of the m42 mutant was slightly impaired when compared to the control viruses. To learn whether a soluble factor released from the macrophages infected with the m42 mutant might be responsible for the growth phenotype, we took supernatants from cultures of bone marrow derived macrophages infected with the m42 mutant, the revertant virus or from untreated cultures at days 0, 3, 6 and 9 p.i. and transferred the filtered supernatants to new cultures that were then infected with MCMVgfp. Compared to medium from uninfected cultures, supernatants taken from infected cultures at days 3, 6 or 9 p.i. impaired virus growth resulting in lower titers (S2A Fig). However, there was no difference whether the supernatants were taken from cultures infected with the m42 mutant or the revertant MCMV. This experiment does not suggest that additional soluble factors (or increased amounts of a factor with antiviral activity) are released from m42-infected cells and are responsible for the reduced replication capacity observed for the m42 mutant.

Myeloid cells have been implicated in defense against MCMV as well as in its dissemination, and thus we asked whether the replication of the m42 mutant is impaired *in vivo* as well. BALB/c mice were infected intraperitoneally with 2 × 10^5^ PFU of MCMVwt, MCMV-m42STOP and MCMV-m42rev, respectively, and viral titers in liver, spleen, lungs and salivary glands were determined on day 3, 7 and 21 p.i. Already on day 3 p.i. titers of the MCMV-m42STOP mutant were markedly reduced, with the median titers ~0.5 to one order of magnitude lower than those of MCMVwt and the rescuant MCMV-m42rev (Fig 3B). Differences of viral titers in the organs were also observed on day 7 p.i. although less pronounced (S2 Fig) and were again obvious when titers were measured on day 21 p.i. in salivary glands (Fig 3B, right panel)–a site where MCMV persists for a prolonged time period.

In an independent experiment, BALB/c mice were infected via the footpad route with the same dose (2 × 10^5^ PFU) of the MCMV-m42STOP and MCMV-m42rev viruses and viral DNA loads in different organs and tissues were determined 3 and 5 days p.i. by quantitative PCR (Fig 3C). In all tissues examined viral loads of the m42STOP mutant were reduced at both time points, confirming the results of the previous experiment. Analysis of transcript levels for viral genes of all three temporal classes (IE, early and late) in the draining lymph node and spleen indicated that the m42STOP mutant was able to establish productive infection and that the viral DNA loads did not simply reflect the inoculated viruses (S2C Fig). The transcript levels for the m42 mutant were lower than those for the rescuant (in spleens for some transcripts below detection limit), again pointing to attenuation as a consequence of the missing m42 gene. Taken together, these results indicate that the m42 gene confers a replication advantage to MCMV *in vivo*, which becomes manifest already on day 3 p.i.

### Expression of the m42 protein precedes CD45 down-modulation

**Fig 4:**
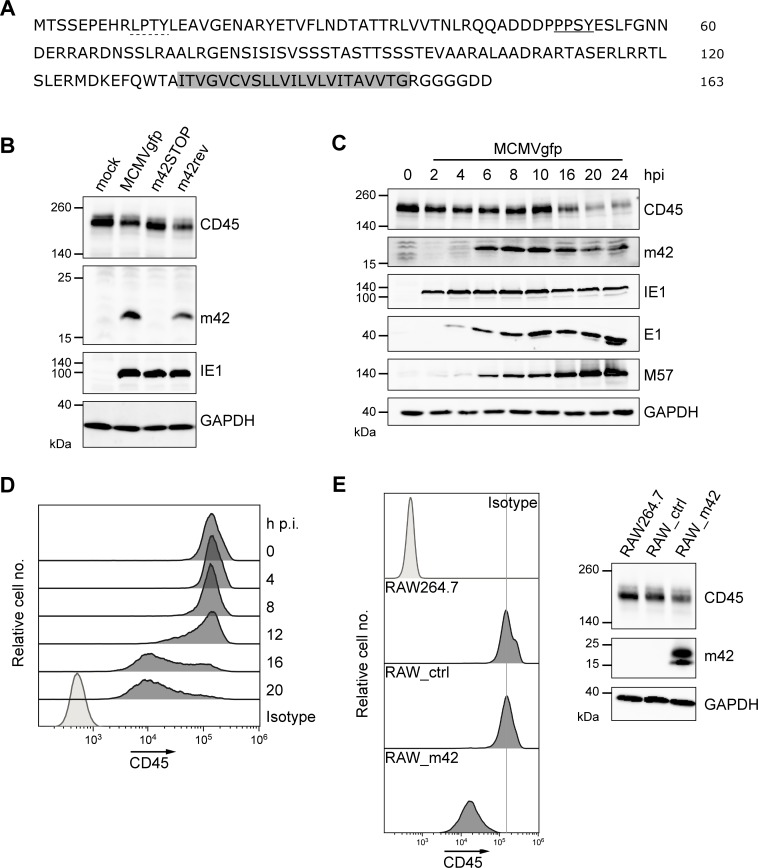
Detection of ORF m42-encoded proteins and kinetics of CD45 down-regulation. (A) Predicted amino acid sequence of the m42 protein with the LPTY (dotted line) and PPSY (underlined) motifs and the putative membrane anchor (gray) marked. (B) RAW264.7 cells were either mock-infected or infected with MCMVgfp, MCMVgfp-m42STOP or MCMVgfp-m42rev, and 24 h p.i. cells were analyzed by immunoblotting for CD45 and m42 expression. IE1 (immediate-early protein 1) served as infection marker and GAPDH as loading control. (C, D) RAW264.7 cells were infected with MCMVgfp, lysed at the indicated time points and expression of CD45 and the viral proteins m42, IE1, E1 and M57 was analyzed by immunoblotting with the respective antibodies. E1, early protein 1; M57, ssDNA-binding protein. (D) In parallel, CD45 surface expression in the MCMVgfp-infected RAW264.7 cells was assessed by flow cytometry. (E, left panel) CD45 surface levels of the parental RAW264.7 cells, the control cell line RAW_ctrl (obtained after transduction with the empty retroviral vector), and the m42-expressing cells were examined by flow cytometry. (right) Expression of CD45, m42 and GAPDH (loading control) in the respective cell lines was determined by immunoblotting.

The polypeptide encoded by ORF m42 is predicted to be a tail-anchored membrane protein with a molecular mass of 17.6 kDa (Fig 4A). In order to detect the protein, a monoclonal antibody (mAb) was generated using a bacterially expressed recombinant protein. In macrophages infected with MCMVgfp or MCMVgfp-m42rev a protein of ~18 kDa specifically reacting with the mAb was observed by immunoblotting, which was absent in cells infected with MCMVgfp-m42STOP (Fig 4B and S1F Fig). In most experiments an additional band with a molecular mass of ~23 kDa was detected, which was often fainter than the 18 kDa band with some variability in abundance (S1B Fig). Consistent with the expected function of m42, the amount of the CD45 protein was decreased in lysates of RAW264.7 cells infected either with MCMVgfp or the rescuant compared to non-infected cells or cells infected with the m42 deletion mutant (Fig 4B). The m42 protein was first detected at 6 h p.i. and expression was strongest between 8 and 16 h p.i., followed by slight decline at later time points (Fig 4C, second panel). The onset of m42 expression was similar to that of the single-strand DNA binding protein M57, indicating that m42 belongs to the early class of viral proteins. When analyzed by immunoblotting, lower amounts of CD45 were observed in the MCMVgfp infected macrophages at 16 h p.i., and the CD45 levels were further decreased at 20 and 24 h p.i. (Fig 4C, top panel). Flow cytometric analysis revealed that the reduction of CD45 surface expression in infected RAW267.4 cells started around 12 h p.i. and was accomplished in the majority of the cells at 16 h p.i., with further progression until 20 h p.i. (Fig 4D). As expected, in cells infected with the m42STOP mutant the CD45 amounts were not diminished during the infection cycle (S1F Fig). Taken together, m42 expression starts early in infection and is followed by CD45 downregulation with a certain temporal delay.

### m42 mediates CD45 down-regulation independent of other viral proteins

The latter result raised the question whether modulation of CD45 surface expression is mediated solely by m42 or whether additional viral proteins are required. To address this point, RAW264.7 cells were transduced with a retroviral vector and a cell line stably expressing m42 was generated (referred to as RAW_m42). The parental RAW264.7 cells and a cell line obtained by transduction with the empty retroviral vector served as controls. Immunoblot analysis confirmed the presence of the 18 kDa as well as the 23 kDa m42 protein in the RAW_m42 cell line, and revealed lower CD45 levels than in control cells (Fig 4E, right panel). In agreement with this result, cell surface expression of CD45 in the RAW_m42 cells was one order of magnitude lower than in the parental RAW264.7 and in RAW_ctrl cells (Fig 4E, left panel), displaying reduction of the CD45 level to a similar extent as in MCMV-infected cells. Thus, m42 expression alone is sufficient for CD45 down-modulation.

### CD45 is internalized from the plasma membrane in cells expressing m42

**Fig 5:**
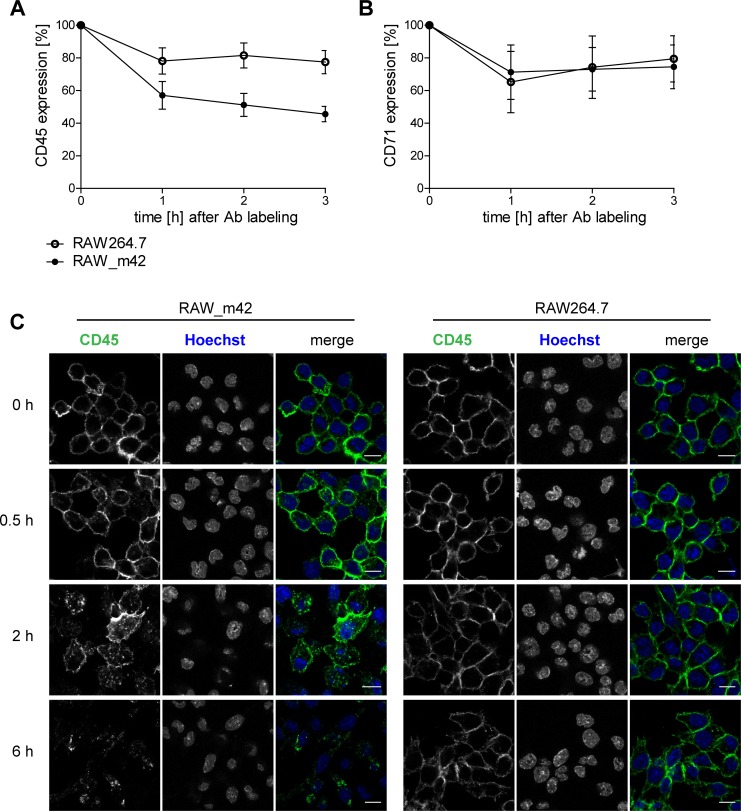
CD45 is internalized from the plasma membrane in m42-expressing cells. (A, B) An internalization assay for CD45 was set up by surface labelling RAW264.7 and RAW_m42 cells with primary Abs directed against CD45 (A) or CD71 (B). Cells were then incubated at 37°C for the indicated time periods, followed by labeling of the primary Ab that remained at the cell surface with corresponding PE-conjugated secondary Abs and flow cytometric analysis. The mean fluorescence intensity of the CD45 and CD71 signals was normalized to the values for time point 0. Results represent mean percentages ± SD of 4 independent experiments. (C) Representative images indicating localization of CD45 in RAW264.7 or RAW_m42 cells after Ab labeling of surface-resident CD45 and cultivation of the cells for the time periods are shown. After fixation and permeabilization of the cells, Ab-labelled CD45 molecules were stained with Alexa647-conjugated secondary Ab and visualized by confocal laser scanning microscopy. Cell nuclei were stained with Hoechst dye. Scale bars, 10 μm.

Possible mechanisms that could explain the m42-mediated CD45 down-regulation are more rapid turn-over of surface-resident CD45 and degradation; however, interference with synthesis, maturation and transport of new CD45 molecules could not be excluded. Metabolic ^35^S-labeling of newly synthesized CD45 protein (pulse-chase experiment) and analysis of the glycosylation pattern of CD45 by treatment with endoglycosidase H did not provide hints that m42 impairs CD45 maturation or transport within the secretory pathway (S3A Fig). This brought the surface-resident CD45 molecules into the focus of our interest and we decided to track them by utilizing a FACS-based internalization assay. After labeling with a CD45 mAb, the RAW_m42 and the parental RAW264.7 cells were kept at 37°C for another 3 h. At various time points CD45 molecules remaining at the plasma membrane were visualized with a PE-conjugated secondary Ab and cells were analyzed by flow cytometry. In normal RAW264.7 cells the amount of labelled CD45 molecules was reduced by 20% within 1 h, but later remained rather constant, possibly indicating internalization and recycling of a fraction of CD45 back to the plasma membrane (Fig 5A). Conversely, in RAW_m42 cells a 40% reduction of the antibody-labelled CD45 molecules at the surface was observed after 1 h and afterwards levels continued to decline. Turnover of the transferrin receptor (CD71) at the cell surface–a protein known to cycle between the plasma membrane and endosomal vesicles–was comparable for both cell lines (Fig 5B), indicating that stronger downregulation of CD45 in RAW_m42 cells was not a general effect applying to other surface proteins. A likely explanation for the observed result is more rapid internalization of CD45 in m42-expressing macrophages. To further substantiate this finding, CD45 present on the surface of the macrophage cell lines was labelled as described above and cells were subsequently fixed and permeabilized at different time points followed by treatment with the fluorescent secondary Ab. Confocal microscopy allowed to detect potentially internalized CD45 molecules as well as those still present at the cell surface. Already 30 min after labelling a fraction of CD45 was found in dot-like structures inside the RAW_m42 cells (Fig 5C). At 2 h the dots appeared more pronounced, and few of the labeled CD45 molecules were still present at the cell surface. This became particularly obvious when compared to the images of the parental RAW264.7 cells, which revealed prominent CD45 surface staining at the time points analyzed and no accumulation of CD45 within the cytoplasm. When examined 6 h post labelling almost no Ab-loaded CD45 was detectable in m42-expressing cells, whereas substantial surface staining was still visible in RAW264.7 macrophages. Similar results were obtained for MCMV-infected cells (S3B Fig). We conclude from these data that in the presence of m42 surface-resident CD45 is rapidly internalized and subsequently degraded, whereas in the parental RAW264.7 cells CD45 is remarkably stable.

### CD45 is degraded via lysosomes involving HECT E3 ubiquitin ligases

**Fig 6:**
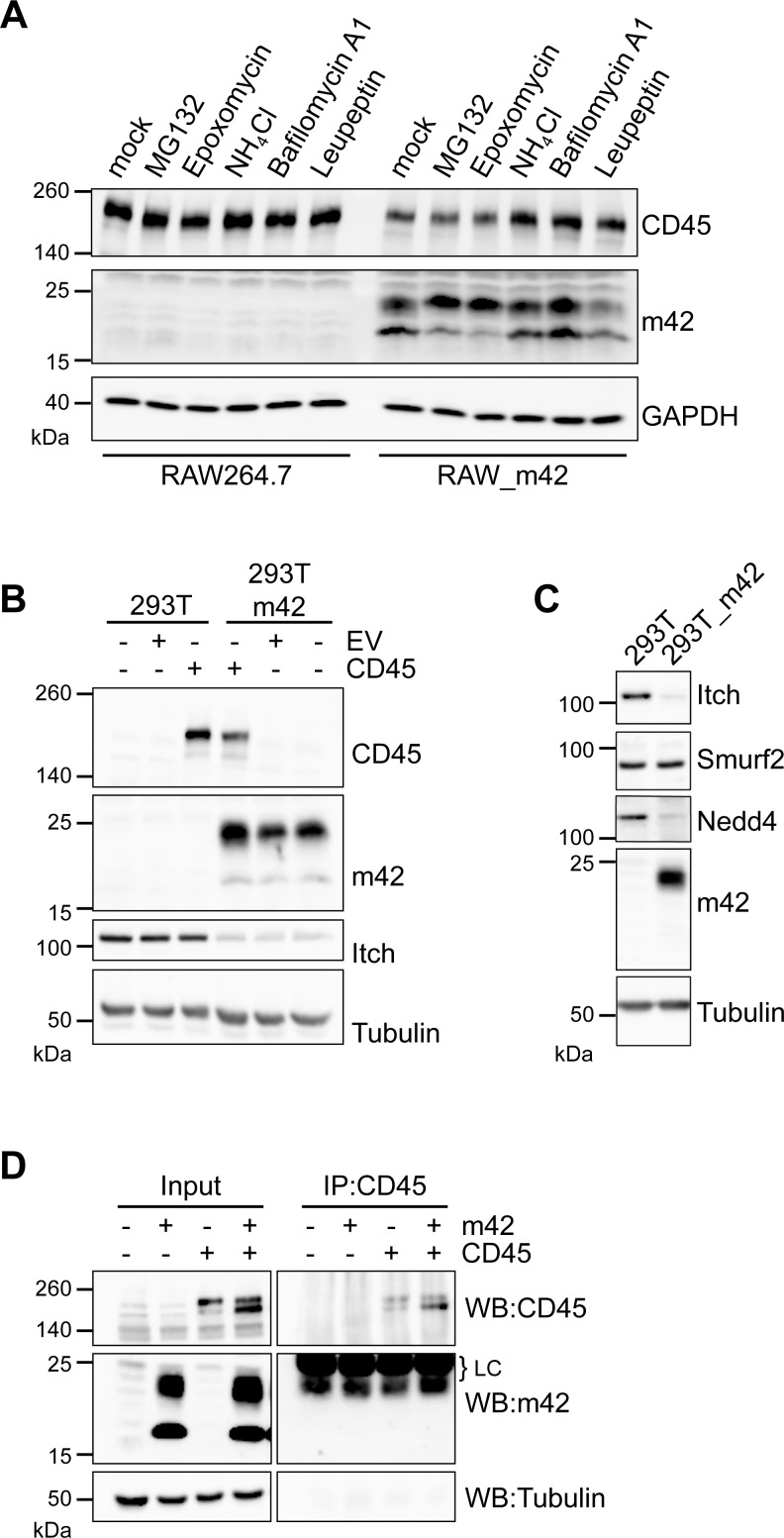
Inhibition of CD45 downregulation in m42-expressing cells by lysosomal inhibitors and interference of m42 with Nedd4-like E3 ubiquitin ligases. (A) RAW264.7 or RAW_m42 were left untreated or were treated with proteasome inhibitors (MG132, Epoxomycin) or lysosome inhibitors (NH_4_Cl, Bafilomycin A1, Leupeptin) for 4 h, followed by immunoblot analysis of CD45 and m42 expression. GAPDH served as loading control. (B, C) HEK 293T cells or a HEK 293T-based cell line stably expressing m42 were either mock transfected or transfected with an empty vector (EV) or a vector encoding murine CD45RB. 48 h post transfection cell lysates were analyzed by immunoblotting for CD45, m42, Itch, Smurf2 and Nedd4 protein expression. α-tubulin served as loading control. (D) HEK 293T cells were transfected with expression vectors for m42 or CD45RB or both vectors and 48 h later cell lysates were subjected to immunoprecipitation with a CD45 antibody. Proteins in lysates and precipitates were detected by immunoblotting using the indicated specific antibodies. LC indicates the signals of the antibody light chain.

To learn which pathway is involved in degradation of CD45, RAW264.7 and RAW_m42 cells were treated with different inhibitors of the proteasome and of lysosomal proteases and cell lysates were subsequently analyzed by immunoblotting for CD45 and m42. In RAW264.7 macrophages CD45 amounts did not change upon treatment with the various inhibitors (Fig 6A), suggesting that CD45 has a long half-life in this cell line. As expected, in RAW_m42 cells CD45 expression was lower than in the parental RAW264.7 cells and was not influenced by treatment with proteasome inhibitors (MG123, epoxomycin). In contrast, when RAW_m42 were exposed to inhibitors affecting the function of lysosomal proteases, CD45 amounts increased (Fig 6A). This result strongly suggests that m42 directs CD45 for degradation via the lysosomal pathway. We also noted that treatment with the proteasome inhibitors increased the abundance of the larger 23 kDa m42 form, whereas the amount of the smaller 18 kDa form was diminished.

Since internalization and degradation of surface proteins often involves ubiquitination [48,49], we searched for hints whether m42 could promote such a mechanism. Analysis of the m42 amino acid sequence did not reveal typical features of an ubiquitin ligase, e.g., a RING domain. Although the m42 protein shares little sequence similarity to the protein encoded by the positional homolog pUL42 of HCMV, the two proteins seem to have some structural properties in common. Both are predicted to be type II transmembrane proteins and possess a short luminal tail. It has recently been reported that the HCMV UL42 protein interacts with Itch [50], a member of the Nedd4-like E3 ubiquitin ligases of the HECT domain family (homologous to the E6AP carboxyl terminus) [51–53]. To examine whether the MCMV m42 protein may employ such a ubiquitin ligase for its activity we performed transfection experiments with HEK 293T cells and a HEK 293T cell line stably expressing m42. In this cell line expression of the HECT E3 ligase Itch was strongly diminished (Fig 6B) and this applied also to Nedd4, but not to Smurf2 (Fig 6C), another member of the Nedd4-like ubiquitin ligases. When the cells were transfected with an expression vector for murine CD45, lower amounts of CD45 were detected in the HEK 293T cells expressing m42 compared to the parental cells (Fig 6B), indicating that the effect of m42 on CD45 expression can be reproduced in this cell line. To test whether there is direct interaction of m42 with CD45, we transfected HEK 293T cells with expression plasmids for m42 and CD45. In this way, higher expression levels for m42 and CD45 could be achieved than in RAW264.7 or HEK 293T cells stably expressing m42 and/or CD45, which should facilitate the detection of a putative interaction. Following immunoprecipitation of CD45 we could not detect co-precipitation of m42 (Fig 6D). Thus, our data do not suggest that there is a direct interaction between m42 and CD45.

**Fig 7:**
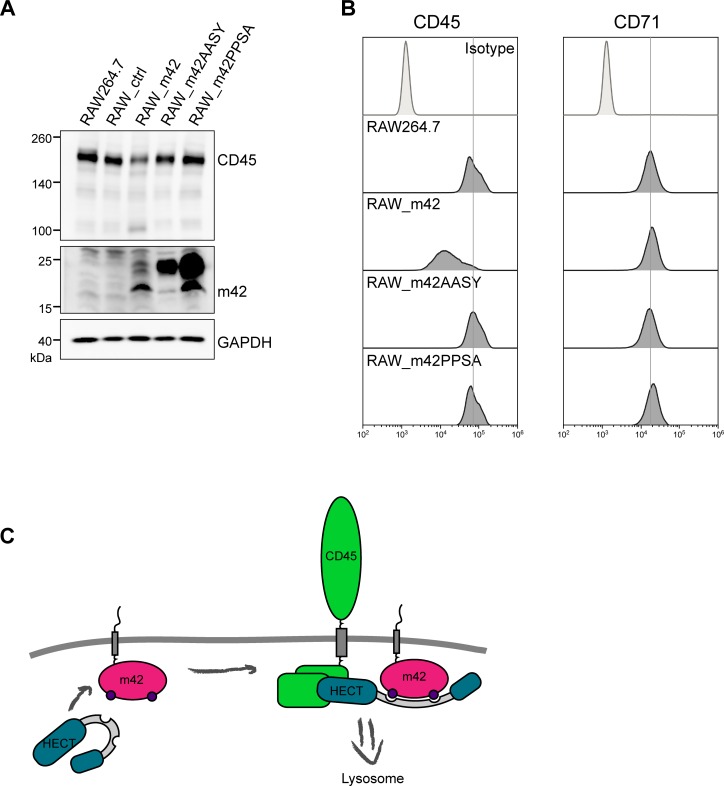
Unaffected CD45 expression in RAW264.7 cell lines expressing m42 variants with a mutated PPSY motif. (A) Analysis of CD45 and m42 levels in RAW264.7 cells stably expressing m42 variants with disrupted PPxY motif. Lysates of RAW264.7, RAW_ctrl, RAW_m42, RAW_m42AASY and RAW_m42PPSA cells were examined by immunoblotting for expression of CD45 and m42. GAPDH served as loading control. (B) CD45 and CD71 surface expression of the indicated cell line was measured by flow cytometry. (C) Proposed model for m42-mediated down-regulation of CD45. By interaction of m42 with Nedd4-like ubiquitin ligases of the HECT family, a conformational change is induced. The thereby activated ubiquitin ligase marks CD45 for internalization and subsequent degradation.

The m42 protein like HCMV pUL42 carries two putative PY motifs (LPTY and PPSY) in the N-terminal part (Fig 4A). Such motifs were found to be relevant for the recruitment of Nedd4-like ubiquitin ligases by HCMV pUL42 [50] and by other related herpesvirus proteins [54]. Since the PPSY motif in m42 is most similar to the respective motifs in HCMV UL42, we introduced two different mutations (AASY, PPSA) into the m42 protein, to learn whether this motif is required for modulation of CD45 expression by m42. Immunoblot and flow cytometric analysis of cell lines stably expressing the m42 variants revealed that CD45 expression was not reduced as in the cells expressing the wildtype m42 protein, and CD45 amounts were comparable to those seen in RAW267.4 cells or in control cells transduced with the empty retroviral vector (Fig 7A and 7B). As expected, surface levels of CD71 were comparable for all cell lines (Fig 7B, right panel). The abundance of the m42 form with the higher molecular mass was markedly increased in the cell lines expressing the proteins with the disrupted PPSY motif (Fig 7A). From this result we concluded that the PPSY motif in m42 is needed for the function of the viral protein to down-modulate CD45, further supporting the notion that a HECT E3 ubiquitin ligase is involved in this process.

## Discussion

In this study we report on the modulation of the surface expression of the cellular protein tyrosine phosphatase CD45 in MCMV-infected macrophages. To our knowledge, such a phenotype has not been described for other viruses or pathogens before, and thus adds a novel element to the arsenal of known virus-host interactions. Here, we focused on the investigation into this phenotype, identified the viral m42 gene as being responsible for mediating the effect, and discovered that CD45 is internalized and degraded in lysosomes, which is most likely induced by m42-mediated activation of Nedd4-like ubiquitin ligases.

The host protein CD45 is expressed abundantly in leukocytes and is essential for keeping signaling pathways of these cells in a primed state [25]. In view of this important function, it may not be surprising that viruses have developed strategies to dampen the activity of CD45 (reviewed in ref. [55]). So far, the only viral proteins reported to interact with CD45, are the HCMV UL11 protein [23] and the E3/49K protein of the species D adenovirus (AdV) type 19a [56]. More recently, it has become clear that many if not all E3/49K homologs of species D AdV possess this property [57] and inhibit the function of T and NK cells via binding to CD45 [56]. Whereas these viral CD45-binding proteins are either secreted or expressed on the surface of infected cells, and are thought to modulate the activity of neighboring immune cells that attack infected cells and threat to eliminate the viruses, the way how MCMV affects CD45 is different in that it acts directly within the infected myeloid cells and thus represents a novel mode of interference with CD45. Compared to lymphocytes, the role of CD45 in myeloid cells is less well analyzed, with only a few publications reporting on an altered cytokine response and adhesion properties of dendritic cells and macrophages lacking CD45 [33,35,37,39,58]. In macrophages completely devoid of CD45 a higher activity of Src family kinases (SFK) such as Hck and Lyn has been observed [33], implying that CD45 negatively regulates these kinases. Analysis of the activity of different SFKs in MCMV-infected macrophages and macrophage cell lines expressing m42 is therefore one of the objectives for further studies. One has to point out, however, that MCMV infection does not lead to a complete loss of CD45 and–according to studies in T cells [59]–variation of CD45 amounts over a wide range may result in only moderate functional consequences.

One effect we observed was the slightly impaired growth kinetics of the m42STOP mutant in bone marrow-derived macrophages, a phenotype which was not visible in murine fibroblasts, pointing to a possible cell-type specific role of m42. We did not find hints that a secreted factor inhibits the growth of the m42 mutant, although in view of the reported CD45-dependent modulation of cytokine production in myeloid cells [35,37,39] this hypothesis was standing to reason. For further analyses a two-pronged approach will be applied; on the one hand pathways downstream of CD45 will be examined, e.g. adhesion of macrophages and cytokine production, and on the other hand—by considering the targeting of Nedd4-like E3 ubiquitin ligases by m42—processes known to be regulated by these ubiquitin ligases will be investigated [51,60].

*In vivo* the m42STOP mutant grew to lower titers in all examined organs and tissues of infected mice. The fact that the replication deficit of the mutant becomes manifest already on day 3 in infected mice is compatible with the idea that m42 interferes with an early defense mechanism. The early innate response has a drastic influence on the amount of viral progeny produced in the first round of replication, setting the stage for the further course of viral infection [61], and such an early effect could therefore also explain the reduced viral titers observed at later time points (e.g., in salivary glands at 21 days p.i.). To which extent the *in vivo* phenotype of the m42STOP mutant is connected with the regulation of CD45 expression remains to be elucidated. Due to the multiple important functions of CD45, particularly in the adaptive immune response, this question cannot easily be addressed by using CD45 knock-out mice. Lack of T cell immunity due to a mutated CD45 gene has for instance been identified as a factor increasing the susceptibility to HSV-1 infection and the risk of herpes simplex encephalitis [62]. The T cell defect in CD45 knock-out mice would mask other, possibly more subtle effects of CD45 in other cell types such as macrophages. Further investigation into the *in vivo* phenotype of the m42 mutant has to await a more comprehensive understanding of the m42-mediated effects at the molecular level.

After identifying the m42 gene we asked by which mechanism the CD45 down-modulation is mediated. We did not detect altered maturation of CD45 in infected macrophages and synthesis of CD45 transcripts was rather increased than reduced (S1E Fig). Although the CD45 protein has been intensively studied over decades, little seems to be known about the turn-over of this molecule. A single publication dating from 1992 [63] reported that interference with glycosylation in the erythroleukemic cell line K562 leads to rapid degradation of newly synthesized CD45 molecules–probably by a pathway that is now known as endoplasmic-reticulum-associated protein degradation [64]. Data from the very same publication suggested that mature CD45 molecules have a long half-life, which is in agreement with our finding that CD45 is quite stable in macrophages as well (Figs 5 and 6A). The internalization assays revealed that in m42-expressing macrophages the internalization rate of CD45 molecules is increased, whereas there was no difference in the internalization of a control protein (transferrin receptor CD71). Moreover, treatment with substances inhibiting the function of lysosomes led to an increase of CD45 levels, indicating that following internalization CD45 molecules undergo lysosomal degradation.

Endocytosis of functionally important surface molecules has been observed upon infection of cells with various viruses, and often involves ubiquitination of the target molecules [65–69]. For the UL42 protein of HCMV it has recently been reported that it interacts with the Nedd4-like E3 ubiquitin ligase Itch and induces its degradation [50]. Although there is little amino acid sequence similarity between pUL42 and the MCMV m42 protein, both of them harbor so-called PY motifs, which are known to mediate the interaction with WW domains, present for instance in E3 ubiquitin ligases of the Nedd4 family [53]. Similar to the findings for HCMV pUL42, we detected here that the amounts of Itch and of the related Nedd4 ubiquitin ligase are severely diminished in cells expressing m42. Interestingly, another member of the Nedd4-like ubiquitin ligases, Smurf2, was not affected, indicating that m42 displays selectivity for certain proteins of this ubiquitin ligase family. Disruption of one of the putative two PY motifs found in m42 led to loss of the ability to regulate CD45 surface expression in macrophages. Based on these data we propose the following model to explain m42-induced internalization and degradation of CD45 (Fig 7C). As has first been suggested for the HSV-2 UL56 protein [70], m42 may act in a similar manner as Nedd4 family-interacting proteins (NDFIPs) that are known to induce conformational changes, thereby releasing Nedd4-like ubiquitin ligases from an auto-inhibitory state. m42 and NDFIPs have indeed structural features in common, including PY-motifs, a transmembrane region and a short C-terminal tail. Once activated, Nedd4 family members can ubiquitinate various substrates [71]. As described for HCMV pUL42 and Itch, interaction triggers degradation of Itch itself [50], and the diminished amounts of Itch and Nedd4 found in m42-expressing cells suggest the same mechanism. It might be that the m42 protein is a substrate for these ubiquitin ligases as well and is turned over together with Itch and Nedd4, because when the PPSY motif is mutated and interaction with the Nedd4-like proteins presumably is disrupted, the abundance of the m42 variants, especially the 23 kDa protein species, was increased. We assume that particularly in this case the 18 kDa m42 version is modified, perhaps by phosphorylation as has been reported for the related UL56 proteins of HSV-2 and equine herpesvirus type-1 (EHV-1) [70,72].

This leaves the question how CD45 could become a substrate for Nedd4-like ubiquitin ligases and whether it is their only target in MCMV-infected cells. Nedd4 family members have been implicated in trafficking of plasma membrane proteins, and substrate specificity is mediated by binding of WW domains to PY motifs as well as other phosphorylated serine and threonine residues in target proteins, or via adapter molecules [73,74]. We could not detect an interaction between CD45 and m42, which argues against an adapter function of m42; however, we cannot exclude that due to the limited detection sensitivity of the m42 antibody this escaped our notion. Interestingly, a potential PY motif (LYSP) is present in the C-terminal intracellular part of murine CD45, which is conserved in human and rat CD45. If an interaction via this PY motif could be experimentally confirmed, this would point to a role of Nedd4-like ubiquitin ligases in the natural turnover of CD45. However, as has been shown for Itch, the interaction between one WW domain and one PY motif is not sufficient for activation [71]. Accordingly, the additional binding of m42 (or of a cellular regulatory protein) may be needed to activate Itch, resulting in the internalization of CD45.

By testing the surface expression of some other proteins (MHC-I, CD40, CD86 and CD71) in macrophages expressing m42 (S4 Fig), we could exclude that m42 increases endocytosis of surface-resident proteins in general. In view of the many known substrate proteins of Nedd4-like ubiquitin ligases [60] it is nevertheless likely that besides CD45, m42 expression affects other specific molecules located at the plasma membrane and also inside the cell, and such substrate proteins are prime candidates for testing in MCMV-infected cells.

In summary, we identified an MCMV gene that interferes with the expression of the host molecule CD45 in myeloid cells, pointing to a new pathway of virus-host interaction. We believe that the mouse model of CMV infection as a natural virus-host system is ideal to further unravel the relevance of the viral down-modulation of CD45 expression. The m42 gene will therefore not only be a powerful tool to learn how MCMV manipulates host pathways to its own advantage, but could also provide us with novel insights into the functions of CD45 in myeloid cells.

## Materials and Methods

### Cells and viruses

The macrophage cell line RAW264.7 (ATCC TIB-71) and the human epithelial cell line HEK 293T (ATCC CRL-3216) were grown in Dulbecco’s modified Eagle’s medium (DMEM) supplemented with 10% FCS, 2 mM glutamine, 100 U/ml penicillin and 100 μg/ml streptomycin. For generation of bone marrow-derived macrophages (BMDM), bone marrow cells were isolated from femurs and tibias of male C57BL/6 mice (purchased from Charles River, Sulzfeld, Germany) as described previously [75] and were kept for 7 days in a mixture of 75% DMEM containing 10% FCS and 25% conditioned medium of L929 cells (ATCC CCL-1) as a source of macrophage stimulating factor [76]. Differentiated cells were routinely tested by flow cytometry and >99% of the cells expressed the macrophage-specific marker F4/80 (Ab BM8; BioLegend). The dendritic cell line DC2.4 [77] was grown in RPMI 1640 medium supplemented with 10% FCS, 2 mM glutamine, 100 U/ml penicillin and 100 μg/ml streptomycin. Murine embryonic fibroblasts (MEF) were prepared from embryos of BALB/c mice (purchased from Charles River) on day 17 of gestation following a published protocol [78] and were grown in the same medium as RAW264.7 cells. Treatment of cells with inhibitors was performed for 4 h with the following substances: MG132 (1.25 μM), epoxomycin (2.5 μM), NH_4_Cl (20 mM) Bafilomycin A1 (50 nM) or Leupeptin (200 μM).

The MCMV strain herein referred to as MCMVwt is derived from the BAC-cloned MCMV genome pSM3fr-MCK-2fl [79]. The strain expressing the enhanced green fluorescent protein (MCMVgfp) and the deletion mutants used in the screen were described previously [44,80]. The genomes lacking the ORFs m42 or M43 were generated in *E*.*coli* using the BAC pSM3fr-GFP and by following the mutagenesis procedure described in Loewendorf et al., 2004. Genomes giving rise to the m42STOP mutant and the m42rev rescuant were once generated for the BAC pSM3fr-MCK-2fl and a second time for a pSM3fr-GFP-derived BAC, in which a frameshift mutation in the m129 locus was repaired as described in Jordan et al. (2011). *En passant* mutagenesis [81] was performed using plasmid pEPkanS2 as template and the following primers to amplify the kanamycin resistance (KanR) cassette: m42STOP_fwd, 5’-ttc cga acc gga gca ccg ttt gcc tac tta tct gga agc ggg cta gtt aac tag ccg tcg gtg aga acg ctc gct agg atg acg acg ata agt agg g-3’; m42STOP_rev, 5’-cgt tga gaa aca cag ttt cat agc gag cgt tct cac cga cgg cta gtt aac tag ccc gct tcc aga taa gta ggc aca acc aat taa cca att ctg att ag-3’. m42rev_fwd, 5’-ttc cga acc gga gca ccg ttt gcc tac tta tct gga agc ggt cgg tga gaa cgc tcg cta gga tga cga cga taa gta ggg-3’; m42rev_rev, 5’-cgt tga gaa aca cag ttt cat agc gag cgt tct cac cga ccg ctt cca gat aag tag gca caa cca att aac caa ttc tga tta g-3’. Viruses based on pSM3fr-MCK-2fl (without GFP gene) were used for *in vivo* experiments. To reconstitute virus mutants, MEF were transfected with the respective BAC-DNA using the jetPEI transfection reagent (Polyplus-transfection, Illkirch, France). Virus stocks were prepared by ultracentrifugation, and viral titers were determined by plaque assay on MEF following established protocols [82]. RAW264.7 macrophages were infected at a multiplicity of infection (MOI) of 3 (if not indicated otherwise) followed by centrifugal enhancement for 30 min at 280 × g.

### Generation of retroviral vectors and stable cell lines

The retroviral vector pLHCX (Clontech) carrying a hygromycin B resistance gene was used to clone the m42 ORF downstream of the HCMV major IE promotor. The PPSY motif was disrupted by site-directed mutagenesis using the retroviral plasmid pLHCXm42 as template and the following primers: AAxY_fwd: 5’-gcc gcat cct acg aga gtc tc-3’; AAxY_rev: 5’-ggg atc gtc gtc cgc-3’ and PPxA_fwd: 5’-gcc gag agt ctc ttt ggt-3’; PPxA_rev: 5’-gga cgg tgg ggg atc-3’. For generation of stable cell lines, retroviruses were produced by transfection of HEK 293T cells with the respective retroviral vector together with plasmids encoding the VSV-G envelope protein and the gag and pol functions, respectively. RAW264.7 cells or HEK 293T cells were transduced with the retroviruses, and 24 h later cells were selected for hygromycin resistance by adding 200 μg/ml Hygromycin B gold (InvivoGen). Cell lines were propagated in medium with 100 μg hygromycin/ml.

### Flow cytometry

Single cell suspensions were incubated for 5 min in 5% horse serum (to block Fcγ-receptors) and subsequently for 20 min at 4°C with antibodies specific for CD45 (clone C363.16A [for experiment shown in Fig 1D] or clone 30-F11 [for remaining experiments]; both eBioscience) or CD71 (R17217; eBioscience). For exclusion of dead cells staining was performed with 7-AAD (10 μg/ml). Subsequently, cells were washed with PBS/2 mM EDTA and fixed with 1.5% PFA. Samples were measured with the Cytomics FC 500 or CytoFLEX Flow Cytometer (both Beckman Coulter) and data were analyzed using Kaluza 1.5 software (Beckman Coulter).

### Immunoblot analysis

Cells were lysed in NP40 lysis buffer (1% NP40, 25 mM Tris-HCl (pH 8.0), 150 mM NaCl, 5 mM EDTA). Following the determination of protein concentrations by Bradford assay (Bio-Rad), the equivalent of 20 μg of total protein (or 60 μg for analysis of m42) was loaded per well on 6–8% SDS-polyacrylamide gels (or 13% for analysis of m42). Antibodies used for probing the blots were CD45 (69/CD45; BD); GAPDH (14C10; Cell Signaling); m42 (m42.02); IE1 (Croma101); E1 (Croma103), M57 (M57.02). Antibodies directed against MCMV proteins were generated and provided by CAPRI (Rijeka, Croatia). Horseradish peroxidase-coupled secondary antibodies (Dako) were used at a 1:5000 dilution. Signals were detected with an LAS-3000 imager following treatment with the ECL Select substrate (GE Healthcare). Images were processed using Adobe Photoshop CS4.

### Immunofluorescence microscopy

Cells were grown on coverslips and either mock-infected or infected with an MOI of 1. At the time points indicated cells were fixed with 3% PFA and permeabilized with 0.2% Triton X-100. Upon blocking with 0.2% gelatin, cells were labelled with the CD45-specific antibody (30-F11; eBioscience) and Alexa Fluor 647-coupled goat anti-rat IgG. Nuclei were counterstained with Hoechst 33342 dye (Cell Signaling). Confocal images were acquired with a Leica DM IRB microscope with a TCS SP2 AOBS scan head and processed using ImageJ and Adobe Photoshop CS4.

### Internalization assay

For the FACS-based internalization assay, cells were labelled with primary antibodies specific for CD45 (30-F11; eBioscience) or CD71 (R17217; eBioscience) for 20 min on ice. After washing cells were incubated at 37°C until samples were cooled on ice at the indicated time points and stained with PE-coupled secondary antibodies, followed by flow cytometric analysis.

For the microscopy-based internalization assay, cells were grown on cover slips. To label surface resident CD45, cells were first incubated on ice for 15 min and then incubated with the 30-F11 antibody for 30 min on ice, followed by washing. Cells were incubated at 37°C and at the time points indicated fixed with 3% PFA. Further treatment was done as described for immunofluorescence microscopy.

### Pulse-chase experiment

Proteins were metabolically labeled by incubating the cells with medium containing (^35^S)-methionine and -cysteine (4–6 MBq per sample) for 30 min. Thereafter, cells were either lysed immediately or further incubated for the indicated time periods in the presence or absence of MG132 (5 μM). Precipitation of CD45 was performed with a specific antibody (30-F11, eBioscience) in combination with protein G-sepharose beads; precipitated proteins were optionally treated with Endoglycosidase H (Roche) and then separated by SDS PAGE. For autoradiography dried gels were exposed to a phosphor screen for 2 weeks and developed using an Optimax machine.

### Infection of mice

Viral replication *in vivo* was analyzed in 7 week-old BALB/c mice upon intraperitoneal infection with 2 × 10^5^ PFU of tissue culture-derived virus stock. At time points indicated mice were sacrificed and viral titers in organ homogenates were determined by plaque assay.

### Quantitation of viral genomes and transcripts

Quantitation of MCMV genomes from organ homogenates were performed as described [83]. Briefly, DNA was isolated using the DNeasy Blood and Tissue Kit (Qiagen) and viral and cellular genomes were quantitated in absolute number by M55-specifc and *pthrp*-specific qPCR normalized to an log_10_-titration of standard-plasmid pDrive_gB_PTHrP_Tdy [84]. Viral transcripts in the draining lymph node were quantified by RT-qPCR specific for m123/IE1, M112/E1 and M86/MCP monitoring all kinetic stages of viral replication as described in greater detail previously [83]. Total RNA was isolated with the RNeasy Mini Kit (Qiagen) and absolute quantification of viral transcripts was performed with graded numbers of the specific in vitro generated transcripts as standard. For normalization cellular ß-actin transcripts were quantitated in parallel. For relative quantification of CD45 mRNA expression in RAW264.7 cells total RNA was isolated with the RNeasy Mini Kit and reverse transcribed into cDNA. qPCR was performed using CD45 specific primer (5´-aga aga gag atc cac cca gtg acc-3´ and 5´-gct cac tct ctt tgc tca tct cca-3´) and CD45 mRNA levels were normalized to expression of β-actin.

### Statistical analysis

Differences between two data sets were evaluated by Student’s t test (unpaired, two-tailed) after log-transformation with Welch’s correction using Graphpad Prism version 5.0 (GraphPad Software, San Diego, CA). *P* values <0.05 were considered as statistically significant.

### Ethic statement

All animal experiments were performed according to the recommendations and guidelines of the Federation of European Laboratory Animal Science Associations (FELASA) and the Society of Laboratory Animals (GV-SOLAS) and animal research protocols were approved by the Niedersächsische Landesamt für Verbraucherschutz und Lebensmittelsicherheit (permission no AZ33.12-42502-04-12/1042), according to German Federal Law ³8 Abs. 1 TierSchG (animal protection law).

## Supporting Information

S1 Fig Downregulation of CD45 surface expression in MCMV-infected macrophage and dendritic cell lines and detection of the 23 kDa m42 protein species. (A) RAW264.7 cells were either mock infected (left) or infected with MCMVgfp (right) at an MOI of 3, and CD45 surface expression was determined by flow cytometry 24 h p.i. 7-AAD staining was used to exclude dead cells and to gate on living cells. (B) RAW264.7 cells were either mock-infected or infected with the indicated viruses and cell lysates harvested 1 day later were subjected to immunoblotting with the m42-specific monoclonal antibody. GFP served as infection marker. (C) Flow cytometric analysis of CD45 expression for RAW264.7 cells 24 h after mock infection (dark filled histogram) or infection with MCMVgfp (gray line) or after treatment with UV-inactivated virus (open histogram). (D) CD45 expression of DC2.4 cells after mock-infection (dark filled histogram) or infection with MCMVgfp (left, open histogram) or MCMV-Δm42 (right, open histogram). For (C) and (D) gating was on living cells and for samples containing infected cells additionally on GFP^+^ cells. (E) CD45 mRNA levels were determined by quantitative RT-PCR for mock-infected and MCMVgfp-infected RAW264.7 cells. (F) RAW264.7 cells were infected with MCMVgfp or MCMV-m42STOP and harvested at the indicated time points, followed by immunoblot analysis with CD45, m42 and IE1 specific antibodies. The asterisk in (B) and (F) mark the 23 kDa m42 species.
(TIF)

S2 Fig Growth analysis of the m42 mutant *in vitro* and *in vivo*, and viral gene expression in lymph nodes and spleens of infected mice. (A) BMDM were either mock-infected (black bars) or infected with MCMV-m42STOP (dark grey bars) or MCMV-m42rev (light grey bars) at an MOI of 0.1. At days 0, 3, 6 and 9 supernatants were harvested and filtered through 0.1 μm syringe filters. MEF seeded in 48 well plates were treated with supernatants 2 h prior to infection with MCMVgfp (20 PFU/well). Depicted are the viral titers in the different cultures at day 7 p.i. as determined by plaque assay. (B) Viral titers at day 7 p.i. in organs of the mice infected as described in Fig 3B. Viral titers of organ homogenates were determined by plaque assay on MEF. (C) Expression of viral transcripts in the draining (popliteal) lymph nodes and in spleens was quantitated in parallel to determination of viral loads (Fig 3C) at days 3 and 5 p.i. by RT-qPCR specific for m123/IE1, m112/E1 and M86/MCP. Data were normalized to 10^8^ cellular β-actin transcripts. Please note that 10 to 100-fold less transcripts were detected for m123/IE1 and m112/E1 compared to M86/MCP (for instance in lymph nodes, for both the mutant and the revertant virus). The number of IE1 and E1 transcripts in spleens at days 3 and 5 p.i. was below the detection limit (10–20 copies). Please note the different scales of the y-axes for the different panels.
(TIF)

S3 Fig Analysis of CD45 maturation and internalization in infected cells. (A) Proteins of RAW264.7 or RAWm42 cells were metabolically labeled with (^35^S)-Met/Cys for 30 min, followed by a chase period of 0, 30 and 90 min. Half of the cultures were treated with 5 μM MG132 during the whole incubation period. At indicated time points cells were lysed and CD45 was immunoprecipitated. One aliquot of each sample was treated with endoglycosidase H. The different forms of CD45 are asterisked. CD45^r^, CD45^s^, and CD45^dg^ denote the EndoH resistant, EndoH sensitive and deglycosylated forms of CD45, respectively. (B) RAW264.7 were seeded on coverslips and infected with MCMVgfp for 24 h. After Ab labeling of surface-resident CD45 cells were incubated for the time periods shown and then fixed and permeabilized. Ab-labelled CD45 molecules were stained with Alexa647-conjugated secondary Ab and visualized by confocal microscopy. Cell nuclei were stained with Hoechst dye. Infected GFP+ cells are encircled with a dotted line and arrows point to cells with intracellular accumulations of CD45. Scale bars, 5 μm.
(TIF)

S4 Fig Expression of other surface molecules is regulated independent of m42 upon MCMV infection. RAW264.7 cells were either mock infected or infected with MCMVgfp or MCMV-Δm42 at an MOI of 3, and CD40, CD86 or MHC class I surface expression were determined by flow cytometry 24 h p.i. 7-AAD staining was used to exclude dead cells and to gate on living cells. For samples with infected cells gating was on GFP^+^ cells.
(TIF)
