# Supplementary figures and images for: The Mouse Cytomegalovirus Gene m42 Targets Surface Expression of the Protein Tyrosine Phosphatase CD45 in Infected Macrophages
Source: PLoS Pathog. 2016 Dec 7;12(12):e1006057. doi: 10.1371/journal.ppat.1006057 (PMC5142792; doi:10.1371/journal.ppat.1006057)

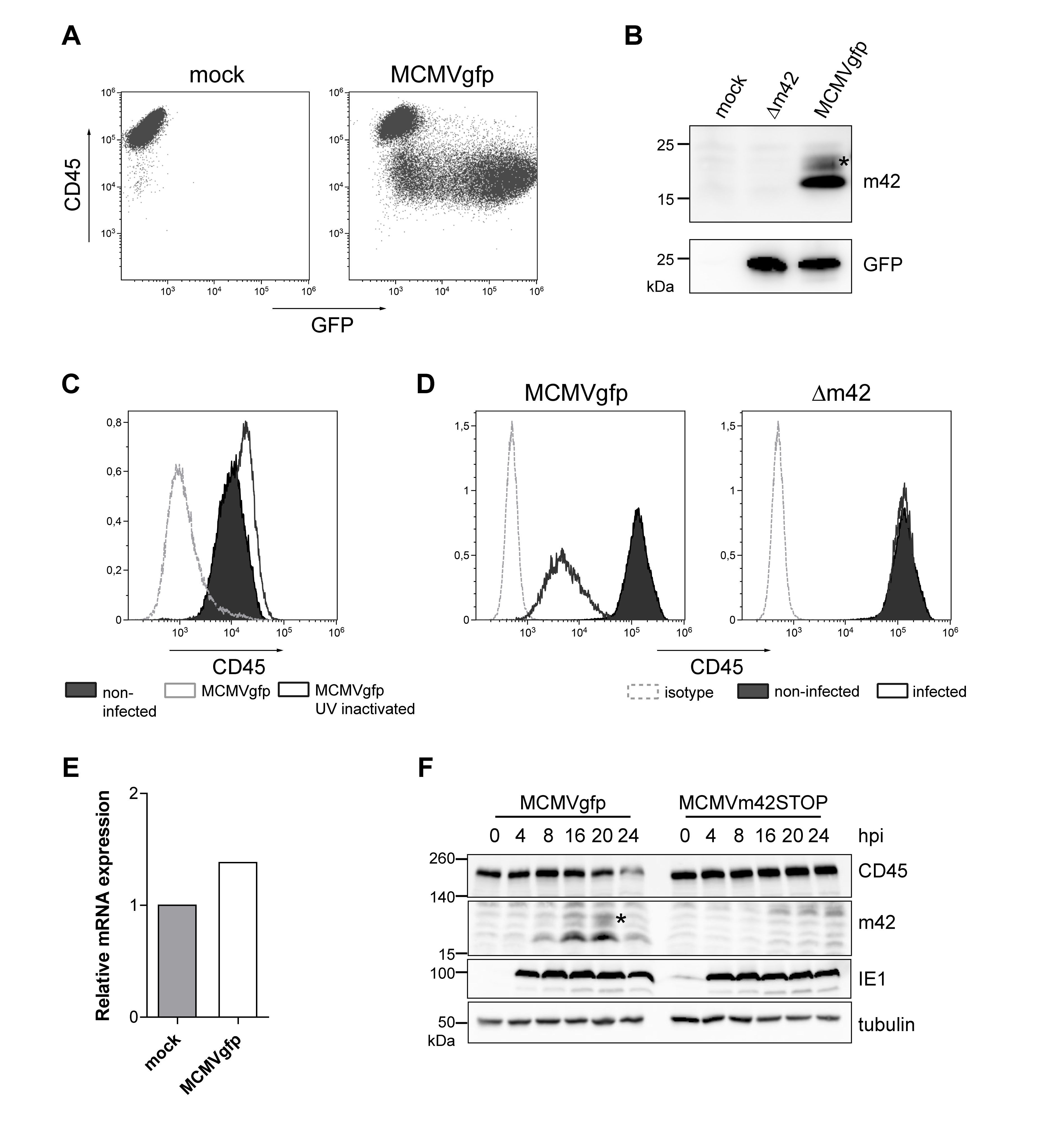

Supplement: S1 Fig — (A) RAW264.7 cells were either mock infected (left) or infected with MCMVgfp (right) at an MOI of 3, and CD45 surface expression was determined by flow cytometry 24 h p.i. 7-AAD staining was used to exclude dead cells and to gate on living cells. (B) RAW264.7 cells were either mock-infected or infected with the indicated viruses and cell lysates harvested 1 day later were subjected to immunoblotting with the m42-specific monoclonal antibody. GFP served as infection marker. (C) Flow cytometric analysis of CD45 expression for RAW264.7 cells 24 h after mock infection (dark filled histogram) or infection with MCMVgfp (gray line) or after treatment with UV-inactivated virus (open histogram). (D) CD45 expression of DC2.4 cells after mock-infection (dark filled histogram) or infection with MCMVgfp (left, open histogram) or MCMV-Δm42 (right, open histogram). For (C) and (D) gating was on living cells and for samples containing infected cells additionally on GFP+ cells. (E) CD45 mRNA levels were determined by quantitative RT-PCR for mock-infected and MCMVgfp-infected RAW264.7 cells. (F) RAW264.7 cells were infected with MCMVgfp or MCMV-m42STOP and harvested at the indicated time points, followed by immunoblot analysis with CD45, m42 and IE1 specific antibodies. The asterisk in (B) and (F) mark the 23 kDa m42 species. (TIF) [file ppat.1006057.s001.tif]

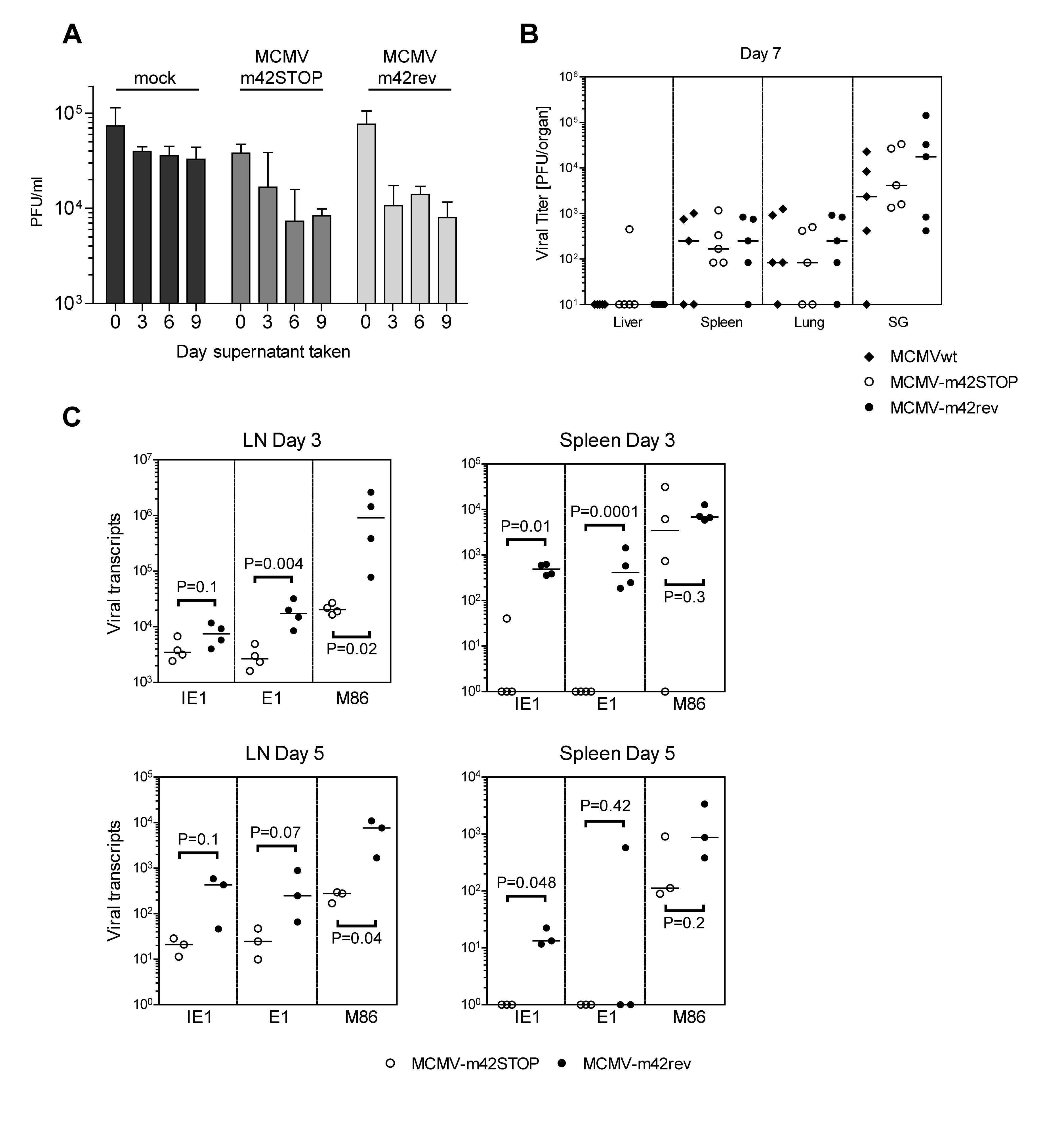

Supplement: S2 Fig — (A) BMDM were either mock-infected (black bars) or infected with MCMV-m42STOP (dark grey bars) or MCMV-m42rev (light grey bars) at an MOI of 0.1. At days 0, 3, 6 and 9 supernatants were harvested and filtered through 0.1 μm syringe filters. MEF seeded in 48 well plates were treated with supernatants 2 h prior to infection with MCMVgfp (20 PFU/well). Depicted are the viral titers in the different cultures at day 7 p.i. as determined by plaque assay. (B) Viral titers at day 7 p.i. in organs of the mice infected as described in Fig 3B. Viral titers of organ homogenates were determined by plaque assay on MEF. (C) Expression of viral transcripts in the draining (popliteal) lymph nodes and in spleens was quantitated in parallel to determination of viral loads (Fig 3C) at days 3 and 5 p.i. by RT-qPCR specific for m123/IE1, m112/E1 and M86/MCP. Data were normalized to 108 cellular β-actin transcripts. Please note that 10 to 100-fold less transcripts were detected for m123/IE1 and m112/E1 compared to M86/MCP (for instance in lymph nodes, for both the mutant and the revertant virus). The number of IE1 and E1 transcripts in spleens at days 3 and 5 p.i. was below the detection limit (10–20 copies). Please note the different scales of the y-axes for the different panels. (TIF) [file ppat.1006057.s002.tif]

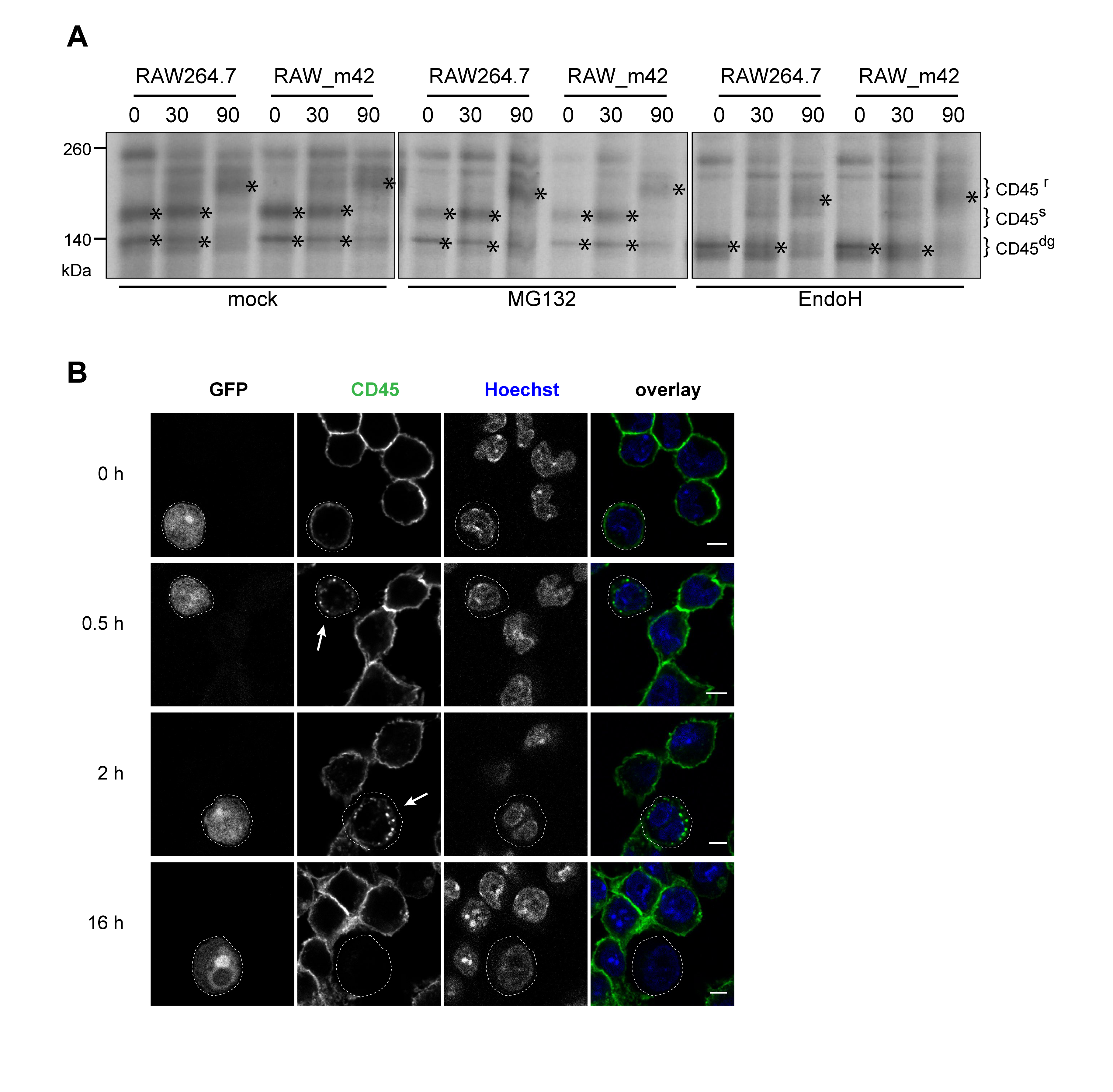

Supplement: S3 Fig — (A) Proteins of RAW264.7 or RAWm42 cells were metabolically labeled with (35S)-Met/Cys for 30 min, followed by a chase period of 0, 30 and 90 min. Half of the cultures were treated with 5 μM MG132 during the whole incubation period. At indicated time points cells were lysed and CD45 was immunoprecipitated. One aliquot of each sample was treated with endoglycosidase H. The different forms of CD45 are asterisked. CD45r, CD45s, and CD45dg denote the EndoH resistant, EndoH sensitive and deglycosylated forms of CD45, respectively. (B) RAW264.7 were seeded on coverslips and infected with MCMVgfp for 24 h. After Ab labeling of surface-resident CD45 cells were incubated for the time periods shown and then fixed and permeabilized. Ab-labelled CD45 molecules were stained with Alexa647-conjugated secondary Ab and visualized by confocal microscopy. Cell nuclei were stained with Hoechst dye. Infected GFP+ cells are encircled with a dotted line and arrows point to cells with intracellular accumulations of CD45. Scale bars, 5 μm. (TIF) [file ppat.1006057.s003.tif]

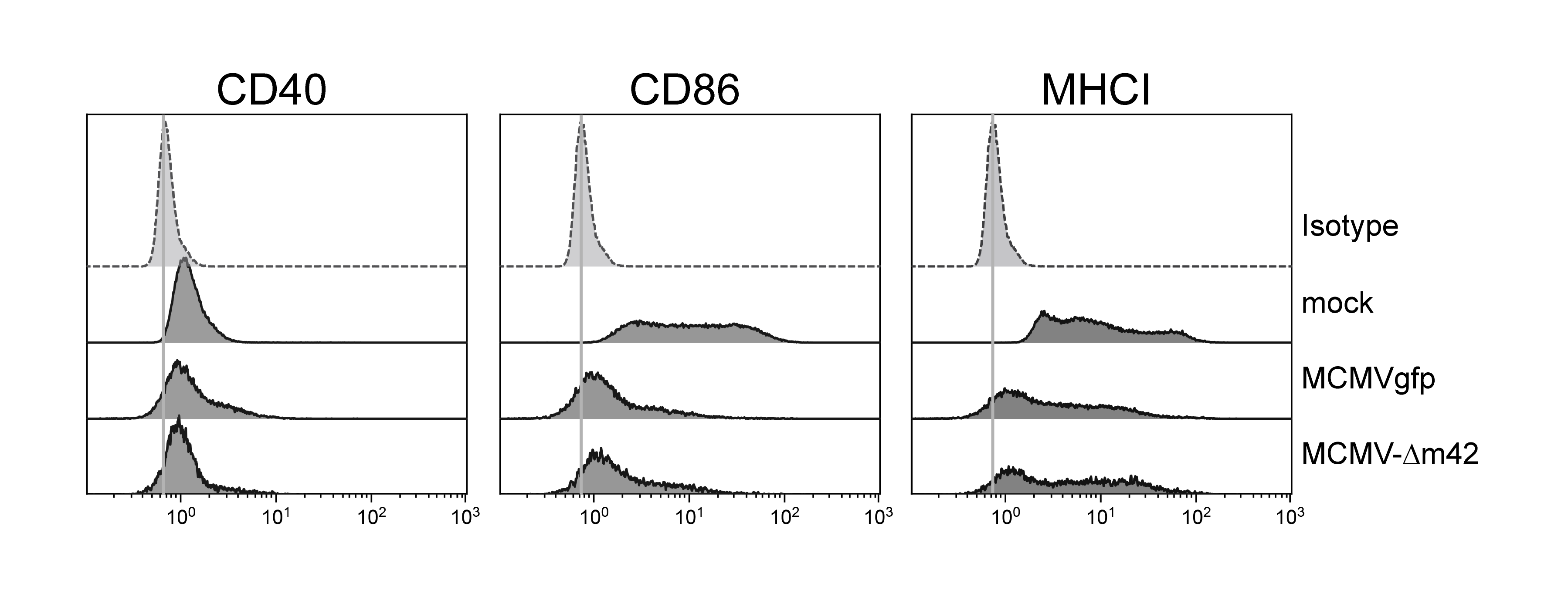

Supplement: S4 Fig — RAW264.7 cells were either mock infected or infected with MCMVgfp or MCMV-Δm42 at an MOI of 3, and CD40, CD86 or MHC class I surface expression were determined by flow cytometry 24 h p.i. 7-AAD staining was used to exclude dead cells and to gate on living cells. For samples with infected cells gating was on GFP+ cells. (TIF) [file ppat.1006057.s004.tif]
